# Impaired adult olfactory bulb neurogenesis in the R6/2 mouse model of Huntington's disease

**DOI:** 10.1186/1471-2202-11-114

**Published:** 2010-09-13

**Authors:** Zacharias Kohl, Martin Regensburger, Robert Aigner, Mahesh Kandasamy, Beate Winner, Ludwig Aigner, Jürgen Winkler

**Affiliations:** 1 Division of Molecular Neurology, University Hospital Erlangen, Erlangen, Germany; 2 Department of Neurology, University of Regensburg, Regensburg, Germany; 3 Institute of Molecular Regenerative Medicine, Paracelsus Medical University, Salzburg, Austria; 4 Laboratory of Genetics, The Salk Institute for Biological Studies, La Jolla, California, USA

## Abstract

**Background:**

Huntington's disease (HD) is an autosomal dominant neurodegenerative disorder linked to expanded CAG-triplet nucleotide repeats within the *huntingtin *gene. Intracellular huntingtin aggregates are present in neurons of distinct brain areas, among them regions of adult neurogenesis including the hippocampus and the subventricular zone/olfactory bulb system. Previously, reduced hippocampal neurogenesis has been detected in transgenic rodent models of HD. Therefore, we hypothesized that mutant huntingtin also affects newly generated neurons derived from the subventricular zone of adult R6/2 HD mice.

**Results:**

We observed a redirection of immature neuroblasts towards the striatum, however failed to detect new mature neurons. We further analyzed adult neurogenesis in the granular cell layer and the glomerular layer of the olfactory bulb, the physiological target region of subventricular zone-derived neuroblasts. Using bromodeoxyuridine to label proliferating cells, we observed in both neurogenic regions of the olfactory bulb a reduction in newly generated neurons.

**Conclusion:**

These findings suggest that the striatal environment, severely affected in R6/2 mice, is capable of attracting neuroblasts, however this region fails to provide sufficient signals for neuronal maturation. Moreover, in transgenic R6/2 animals, the hostile huntingtin-associated microenvironment in the olfactory bulb interferes with the survival and integration of new mature neurons. Taken together, endogenous cell repair strategies in HD may require additional factors for the differentiation and survival of newly generated neurons both in neurogenic and non-neurogenic regions.

## Background

Huntington's disease (HD) is a devastating autosomal dominant hereditary neurodegenerative disease caused by a CAG trinucleotide repeat expansion within the *huntingtin *gene encoding an extended polyglutamine tract in the huntingtin (htt) protein [1]. The progressive clinical phenotype consists of involuntary choreic movements, cognitive decline and psychiatric symptoms (reviewed in [2]). Impaired olfactory function was also observed in patients as well as presymptomatic gene carriers [3,4]. Emerging evidence suggests that mutant htt leads to selective neuronal damage by a gain of toxic function inducing neuronal loss and gliosis predominantly in the neostriatum and the cortex (reviewed in [5]).

The presence of neural stem cells in the adult central nervous system (CNS) has been widely described both in rodents and in humans (reviewed in [6]). In adult mammals these cells are present in neurogenic regions of the CNS, the subventricular zone (SVZ) of the lateral ventricle wall and the dentate gyrus (DG) of the hippocampus. New cells are continuously generated in the SVZ [7,8] and physiologically migrate via the rostral migratory stream (RMS) to the olfactory bulb (OB) [9,10]. Here they mainly differentiate into GABA-ergic interneurons in the granular cell layer (GCL) or to a lesser extent in the glomerular layer (GLOM), and functionally integrate [11,12]. New GLOM neurons are further subdivided based on their immunoreactivity against tyrosine hydroxylase (TH), calbindin or calretinin [13]. Neurogenesis in the OB is a life long phenomenon in the mammalian brain, leading to an increase in the total number of neurons in the GLOM, while the number of neurons in the GCL appears to be counterbalanced by continuous cell death [14]. Importantly, neurogenesis in the adult CNS can be modulated by various molecular and environmental factors, in part acting differentially on both neurogenic regions [6].  The stimulation of this endogenous pool of immature neurons may be a useful tool for a cell-based therapeutic approach in neurodegenerative disorders [15].

In transgenic animal models of HD impaired adult neurogenesis has been described in the DG of the hippocampus [16,17], which could be partially restored by environmental stimuli or antidepressants [18,19]. In contrast, the proliferation of neural stem cells in the SVZ is not affected in transgenic HD mouse models [20,21]. The R6/2 HD mouse model carries exon 1 of the human HD gene with an expanded CAG trinucleotide repeat strand (130-150 CAG repeats; [22]) and exhibits decreased striatal and total brain size, ubiquitinated nuclear and cytoplasmic inclusion bodies [23], and progressive motor and cognitive deficits [24]. These mice have an early onset of symptoms associated with a fast progression and a limited life span of approximately 12 to 17 weeks, depending on the colony (reviewed in [25]).

To further rule out the impact of mutant htt on the endogenous generation of new neurons we investigated the SVZ/OB system in the R6/2 mouse model and analyzed the survival and differentiation of SVZ-derived new neurons migrating towards their physiological target region, the OB, and to the adjacent severely affected striatum.

## Results

### Aggregates of mutant huntingtin appear in mature neurons, but not in neuroblasts in R6/2 mice

**Figure 1:**
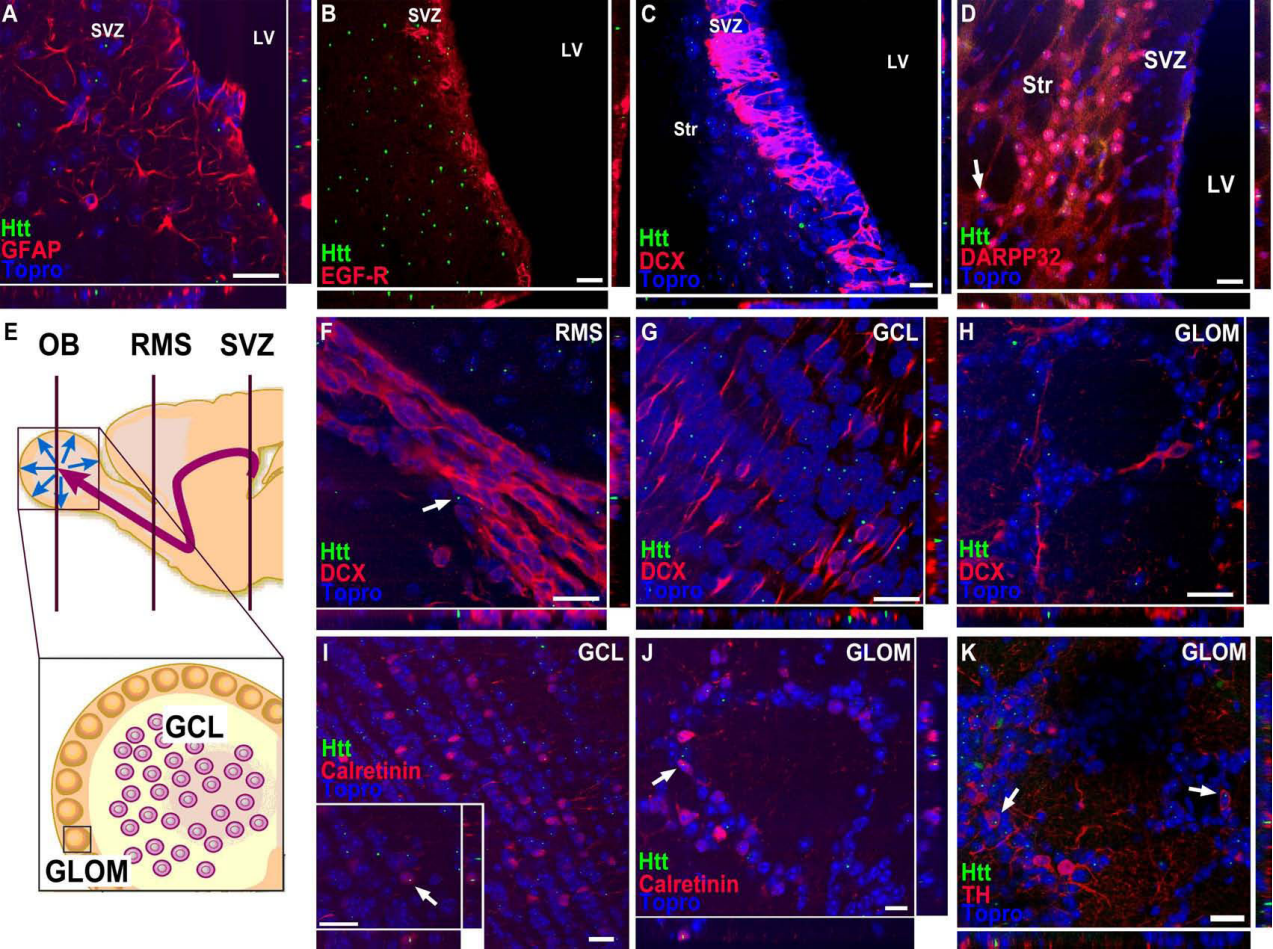
**Expression pattern of mutant huntingtin in developing and mature neurons of the subventricular zone and the olfactory bulb in R6/2 mice**. SVZ derived neurons originate from dividing GFAP expressing cells ("stem cells", B cells) that generate transit-amplifying cells (C cells) which in turn give rise to neuroblasts (A cells). DCX-expressing neuroblasts migrate along the RMS to the olfactory bulb and differentiate into different neuronal cell types in the GCL and the GLOM. Schematic representations of regions of adult neurogenesis in the SVZ/olfactory bulb (**E**, adapted from (35)). Aggregates of mutant huntingtin (green) are absent in GFAP expressing SVZ stem cells (red, **A**), EGF-R expressing transit-amplifying cells (red, **B**) and neuroblasts with DCX immunoreactivity (red, **C**), but appear in DARPP-32 positive mature neurons of the adjacent striatum (red, **D**) of R6/2 mice. Moreover, neuroblasts of the RMS (**F**), the GCL (**G**) and the GLOM (**H**) do not show immunoreactivity for mutant huntingtin. In contrast, aggregates of mutant huntingtin (green) were detected in mature, calretinin-expressing neurons (red) of the GCL (**I**) and the GLOM (**J**) and in TH expressing neurons of the GLOM (red, **K**) of the olfactory bulb. Topro-3 (blue) was used as counterstain for cell nuclei. Scale bar represents 20 μm.

To determine whether aggregates of mutant htt were present in the SVZ/OB (Fig. 1E), we used an α-huntingtin antibody specific for the N-terminus of human mutant htt with a repeat expansion > 82 repeats (MAB5374; [26]). We detected intranuclear immunoreactivity in some mature calretinin-expressing neurons of both the GCL and the GLOM (Fig. 1I,J) and in TH-expressing neurons of the GLOM (Fig. 1K). In contrast, mutant htt was not detected in doublecortin (DCX)-expressing neuroblasts of R6/2 animals in the GCL and the GLOM of the OB (Fig. 1G, H), or in migrating DCX positive neuroblasts in the RMS (Fig. 1F) and the SVZ (Fig. 1C). Besides the DCX expressing SVZ neuroblasts (or A cells) neither glial fibrillary acidic protein (GFAP) expressing B cells (Fig. 1 ) nor epidermal growth factor (EGF)-receptor expressing C cells (Fig. 1) showed intranuclear immunoreactivity for htt aggregates. In contrast, mature neurons of the adjacent striatum expressing dopamine- and cAMP-regulated phosphoprotein-32 (DARPP-32) contained aggregates of htt (Fig. 1D). No immunoreactivity of mutant human htt was observed in the SVZ/OB of WT mice (data not shown).

### Increased migration of neuroblasts towards the striatum in R6/2 mice

**Figure 2:**
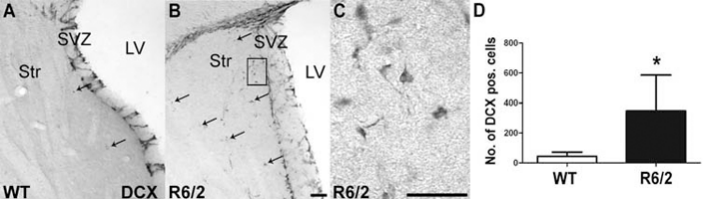
**R6/2 mice show increased migration of neuroblasts to the affected striatum compared to controls, without signs of substantial striatal neurogenesis**. Representative images of DCX-expressing neuroblasts in wild-type (WT; **A**) and R6/2 mice (R6/2; **B**), higher magnification (from insert in **B**) of cells exhibiting heterogeneous morphologies (**C**): Some cells possess only short or no processes, some display multiple well-developed processes characteristic of migrating cells. Arrows indicate migrating DCX positive cells leaving the SVZ. Quantification of DCX-positive cells in the striatum of WT and R6/2 mice, predominantly in close proximity to the SVZ (**D**). Error bars represent SD, * indicates p < 0.05. Scale bars represent 50 μm.

We observed an increased number of neuroblasts in the striatum adjacent to the SVZ. Quantification of DCX-positive cells in the striatum revealed significantly higher numbers in transgenic animals compared to WT (44.0 ± 28.1 in WT vs. 348.0 ± 237.7 in R6/2, p < 0.05, Fig. 2D). These cells were predominantly located in close proximity to the SVZ with a decline of cell number away from the SVZ (Fig. 2A-C). This may be due to neuroblasts migrating into the striatum. At distances greater than 300 μm from the SVZ no DCX-immunopositive neuroblasts were observed. To investigate if any new cells obtained a mature neuronal phenotype in the striatum, we investigated possible colocalization of bromodeoxyuridine (BrdU) with the mature neuronal marker NeuN. In contrast to a previous study [20], we did not observe BrdU/NeuN positive cells 4 weeks after BrdU treatment. Moreover, as none of the DCX-positive striatal cells colabeled with BrdU, we excluded that the lack of striatal neurogenesis was due to a delayed maturation longer than 4 weeks. These results in line with previous studies [21,27] suggest that neuronal maturation of newly generated cells is not present within the R6/2 striatum of 9-week old mice.

### SVZ cell proliferation not affected in R6/2 mice

**Figure 3:**
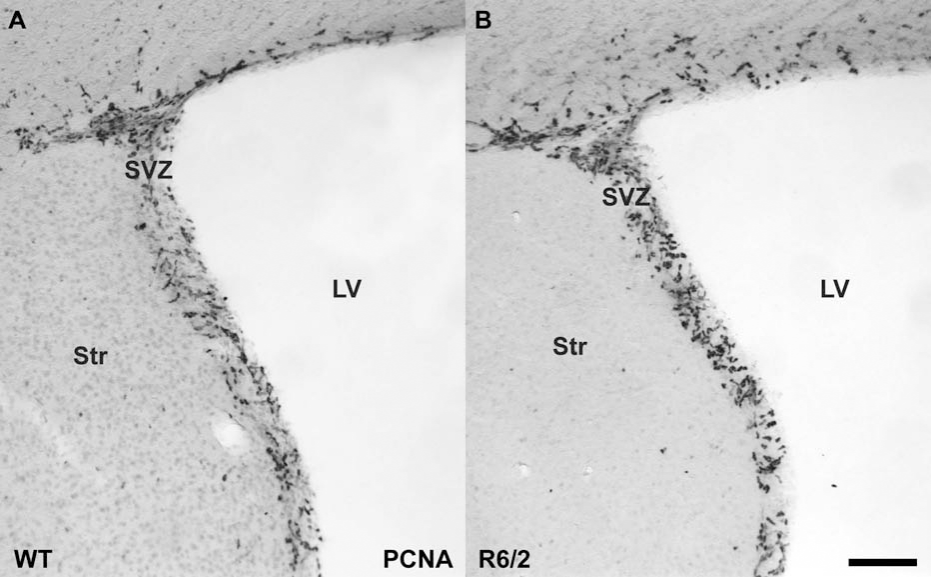
**Cell proliferation in the subventricular zone of the R6/2 HD animals is unchanged compared to WT controls**. Staining for dividing cells with the cell cycle marker PCNA revealed no difference in the amount of stem and precursor cell proliferation in the SVZ of WT (**A**) and R6/2 animals (**B**). Scale bar represents 20 μm.

Proliferation in the SVZ was assessed by quantification of cells labeled for proliferating cellular nuclear antigen (PCNA). In accordance with previous data [21,28] the number of proliferating cells in the SVZ of R6/2 animals was unchanged compared to WT (8.49 ± 2.16 vs. 7.42 ± 3.11 (×10^3^); Table 1, Fig. 3).

**Table 1:** Quantification of adult neurogenesis in the olfactory bulb of wildtype and R6/2 mice

	WT	R6/2
Proliferation in the SVZ		
PCNA (×10^3^)	7.42 ± 3.11	8.49 ± 2.16
		
Neurogenesis in the OB granular cell layer		
BrdU (×10^3^)	69. 9 ± 15.1	46.8 ± 11.3 ^#^
% BrdU/NeuN	88 ± 4	87 ± 3
BrdU × % BrdU/NeuN (×10^3^)	61.5 ± 15.1	40.7 ± 11.2 ^#^
		
Neurogenesis in the OB glomerular layer		
BrdU (×10^3^)	10.1 ± 3.9	4.8 ± 1.7 ^# ^
% BrdU/NeuN	90 ± 3	90 ± 6
BrdU × % BrdU/NeuN (×10^3^)	9.1 ± 3.5	4.4 ± 1.7 ^#^
		
% BrdU/TH	8 ± 2	14 ± 3 ^#^
BrdU × % BrdU/TH	775 ± 301	656 ± 270

Morphological quantification of neurogenesis in the olfactory bulb of WT and R6/2 mice comprises the following measures: total number of proliferating cells in the subventricular zone (PCNA), total number of surviving newly generated cells (BrdU), percentage of newly generated neurons (% BrdU/NeuN) and total number of new neurons (BrdU × % BrdU/NeuN) in the granular cell layer. For the glomerular layer, total number of surviving newly generated cells (BrdU), percentage of newly generated neurons (% BrdU/NeuN), percentage of newly generated dopaminergic neurons (% BrdU/TH), total number of new neurons (BrdU × % BrdU/NeuN) and total number of new dopaminergic neurons (BrdU × % BrdU/TH) are indicated. All measures are expressed as mean ± SD. ^# ^indicates significance comparing WT vs. R6/2 (p < 0.05).

### Decreased olfactory neurogenesis in HD mice

**Figure 4:**
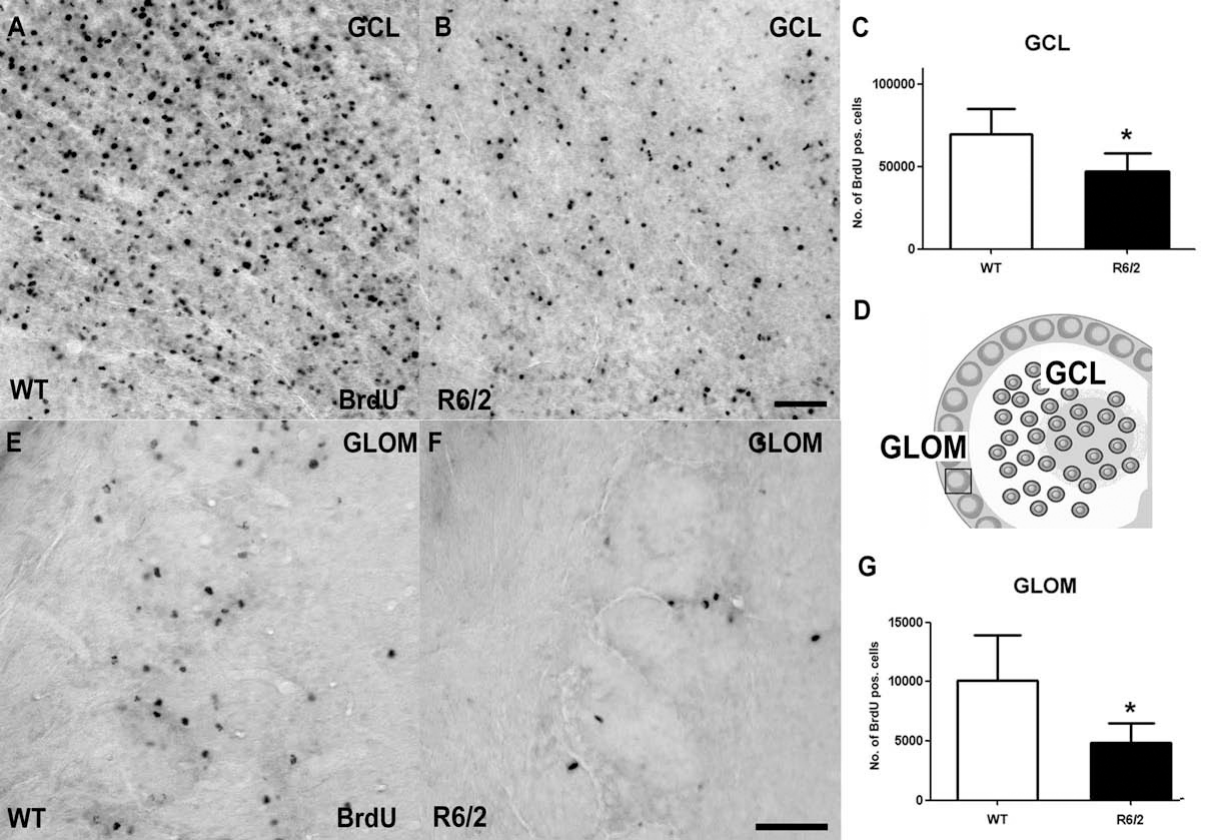
**Impaired survival of newly generated cells in the two neurogenic regions of the olfactory bulb in HD mice compared to controls**. Significant reduction of BrdU-labeled newly generated cells in the GCL and the GLOM 4 weeks after BrdU administration in R6/2 mice compared to controls (**C**, **G**). Representative images of immunostainings for BrdU in the GCL (**A**, **B**) and the GLOM (**E**, **F**) of both groups. Schematic representation of the OB areas analyzed (**D**). Error bars represent SD, * indicates p < 0.05. Scale bars represent 50 μm.

**Figure 5:**
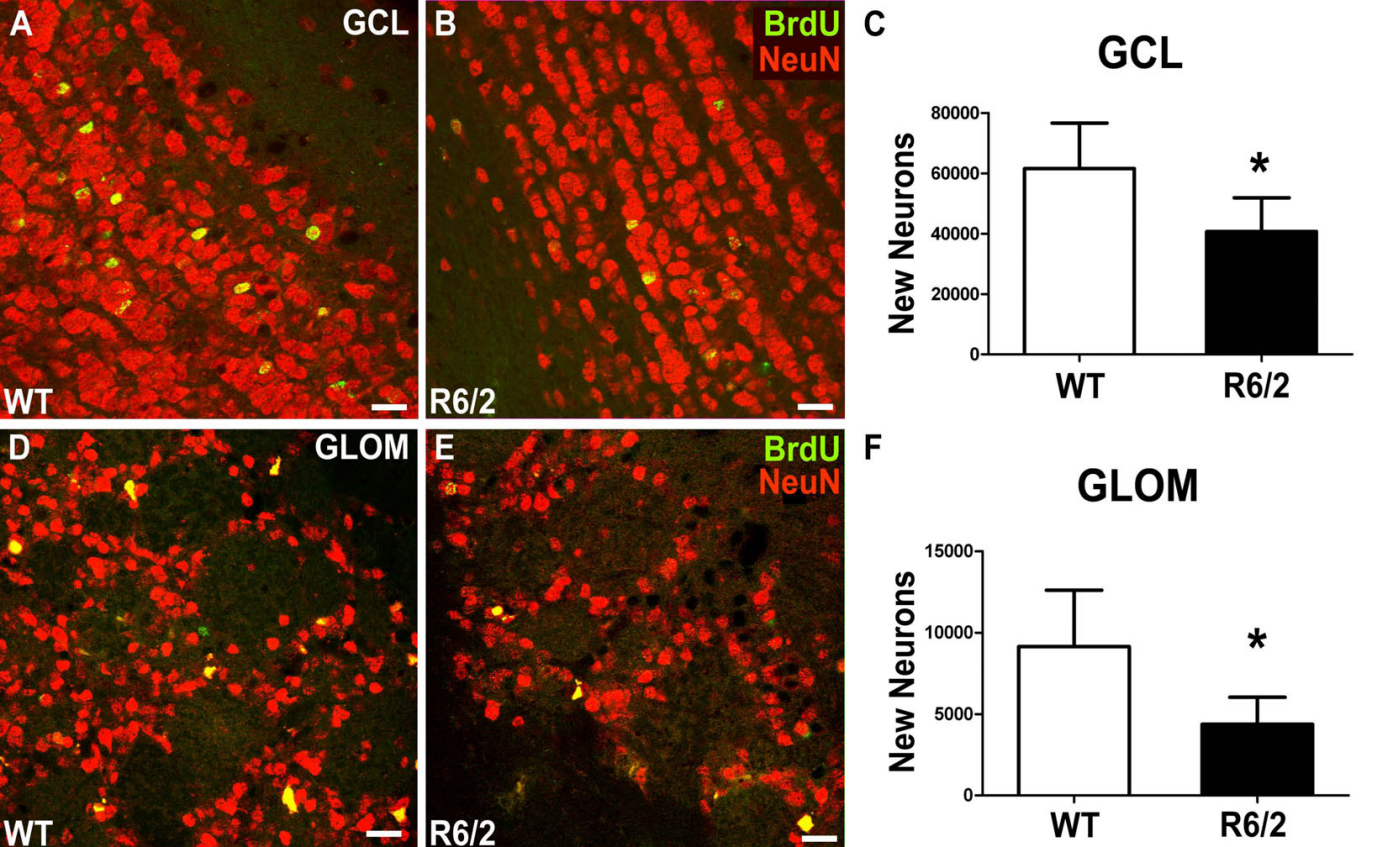
**The generation of new neurons in the neurogenic regions of the olfactory bulb is impaired in R6/2 animals compared to controls**. In the GCL the percentage of newly generated cells that differentiate into a neuronal phenotype is unchanged comparing WT and R6/2 animals, as determined by double-labeling for BrdU and NeuN (**A**, **B**). Calculation of absolute numbers of surviving new neurons in the GCL revealed significantly decreased neurogenesis in HD mice compared to controls (**C**). Analysis of differentiation in the GLOM demonstrated unchanged neuronal differentiation in this region as well. Calculation of the total number of newly generated neurons in the GLOM showed a reduction of more than 50% in R6/2 mice compared to WT controls (**F**). **D **and **E **show representative immunostainings for BrdU and NeuN in the GLOM. Error bars represent SD, * indicates p < 0.05. Scale bars represent 20 μm.

To study the amount of newly generated cells in the OB which showed no structural abnormalities we analyzed the number of BrdU-labeled cells in the GCL and in the GLOM separately. For further investigation, the neuronal identity of these newly generated cells was determined by double-labeling with BrdU and NeuN. The total number of newly generated BrdU/NeuN double-labeled cells was determined to evaluate the effect of mutant htt on OB neurogenesis. In the GCL we observed a significant 33% reduction of BrdU-positive cells in R6/2 animals compared to the WT group (46.8 ± 11.3 vs. 69.9 ± 15.1 (×10^3^); p < 0.05; Table 1, Fig. 4A-C). In the GCL no significant difference in neuronal differentiation was detected (87% vs. 88%, p > 0.05; Table 1). Due to the considerable reduction in newly generated cells this resulted in a significant decrease in total BrdU/NeuN cell numbers by 33% (40.7 ± 11.2 vs. 61.5 ± 15.1 (×10^3^); p < 0.05; Table 1; Fig. 5A-C).

**Figure 6:**
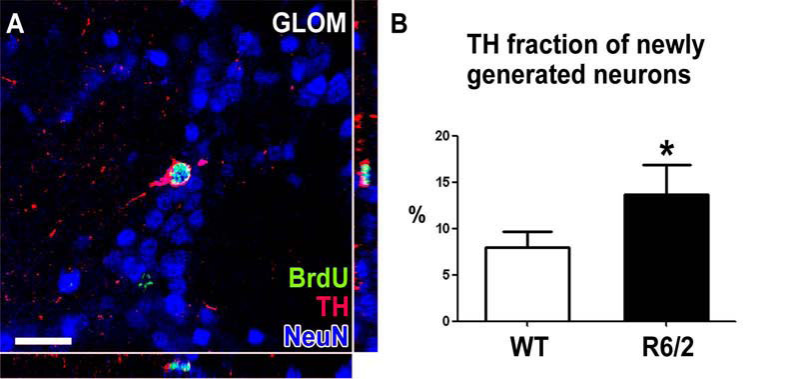
**Generation of dopaminergic neurons in the glomerular layer is less severely affected in HD mice compared to WT aminals**. Further analysis of the phenotype of newly generated neurons in the GLOM of the olfactory bulb revealed that the proportion of new dopaminergic neurons (labeled for BrdU, NeuN and TH, **A**) was significantly increased in HD mice compared to controls (**B**). Scale bar represents 20 μm.

In the GLOM, an even more pronounced 52% reduction of BrdU positive cells was measured in the R6/2 group compared to WT (4.8 ± 1.7 vs. 10.1 ± 3.9 (×10^3^); p < 0.01; Table 1, Fig. 4E-G). As in the GCL, differentiation into a neuronal phenotype remained unchanged (91% vs. 90%, p > 0.05; Table 1). The resulting number of newly generated neurons was significantly reduced in R6/2 mice compared to WT mice (4.4 ± 1.7 vs. 9.1 ± 3.5 (×10^3^); p < 0.05; Table 1; Fig. 5D-F). Interestingly, the fraction of BrdU-labeled newborn cells that also expressed TH as a marker of a dopaminergic phenotype was elevated in the R6/2 group (14 vs. 8%; p < 0.05; Table 1; Fig. 6A, B). This led to the finding that the total number of newly generated dopaminergic GLOM neurons was unchanged comparing R6/2 with WT animals (656 ± 270 vs. 775 301; p > 0.05; Table 1).

### Increased apoptosis in the OB of R6/2 mice

**Figure 7:**
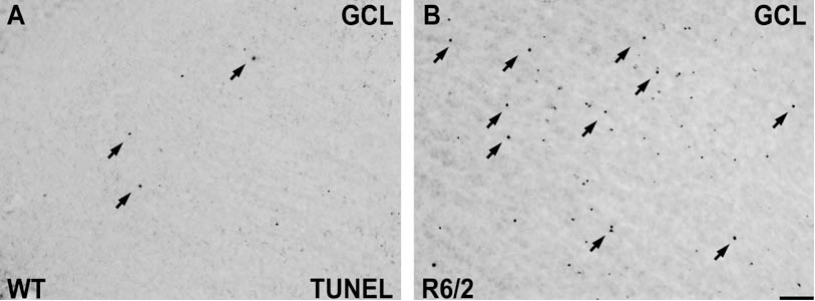
**Increased cell death in the olfactory bulb of R6/2 mice**. TUNEL staining of the GCL in the OB of WT and R6/2 animals shows an increase of TUNEL-positive profiles in transgenic animals carrying the mutant huntingtin (**B**) compared to WT mice (**A**). Scale bar represents 50 μm.

An increase of cell death has been described in the cortex and the striatum of R6/2 mice [29]. To examine if increased cell death is, at least in part, attributable to the reduction in adult neurogenesis in the OB of these HD animals, we performed immunohistochemical staining for terminal deoxynucleotidyltransferase-mediated dUTP nick-end labeling (TUNEL)-positive cells in the OB. We found a substantial increase of TUNEL labelled cells in the GCL of R6/2 mice compared to WT mice (Fig. 7A, B). This indicates that there is increased cell death in the presence of mutant htt in the OB.

## Discussion

Our data provide evidence that the expression of mutant human htt with a CAG repeat expansion in transgenic R6/2 mice is associated with a significant impairment in the survival of newly generated neurons in the OB. We demonstrated that both neurogenic regions of the OB - the GCL and the GLOM - are severely affected in this HD model. Interestingly, mutant htt could not be detected in migrating neuroblasts of the SVZ/OB system or in cells of earlier stages of neural development in the SVZ (B and C cells). In contrast, numerous mature neurons in the striatum and OB contain aggregates of mutant htt. Thus, impaired adult neurogenesis in the OB may be caused by non cell-autonomous mechanisms. Furthermore, it is noteworthy that it remains unclear whether htt aggregates have a potential neuroprotective or toxic function [30]. While there are recent studies on aggregation kinetics of mutant htt and its different fragments [31,32], htt aggregration in developing neurons remains elusive.

Adult neurogenesis in the SVZ/OB system is a highly regulated process consisting of proliferation of neural precursor cells in the SVZ [33] and the subsequent maturation into neuroblasts that migrate along the RMS to the OB. While hippocampal neurogenesis is reduced in the R6/2 mouse model [16,21,28], we confirmed previous studies that the proliferation of neural precursors in the SVZ of R6/2 animals is unchanged [20,21]. Since mutant htt severely affects the adjacent striatum, one potential repair strategy to repopulate this region is to redirect SVZ-derived neuroblasts to the striatum. Redirection of migrating neuroblasts to a lesion site was first described in an animal model of cerebral ischemia [34]. In a model of Parkinson's disease, the combined application of growth factors such as epidermal growth factor and fibroblast growth factor 2 was sufficient to enhance migration of neuroblasts to the striatum; however it did not result in neuronal maturation [35]. In the present study, we observed significantly more cells expressing the neuroblast marker DCX in the striatum of R6/2 mice. These neuroblasts are localized in close proximity to the SVZ, indicating that these precursor cells may migrate as neuroblasts into the diseased R6/2 striatum. Using colocalization of BrdU with NeuN and of BrdU with DCX, we showed that these neuroblasts do not acquire a mature neuronal phenotype. Striatal neurogenesis in HD animal models was previously assessed with conflicting findings: The number of DCX-expressing striatal cells was increased in quinolonic acid-lesioned animals [36], and was even more pronounced when neural and mesenchymal stem cells were transplanted into the lesioned striatum [37]. On the other hand, no alterations in DCX-expressing cells were observed in untreated transgenic R6/2 mice compared to WT controls [21]. Moreover, another study revealed the presence of BrdU/NeuN double-positive cells in the striatum of R6/2 animals [20], which we could not confirm with our present findings. Furthermore, the intraventricular application of combined brain-derived neurotrophic factor and Noggin was effective to generate significant numbers of BrdU-labeled mature striatal neurons expressing DARPP-32 and glutamic acid dehydrogenase-67 (GAD67) in R6/2 mice [27] indicating that additional neurotrophic factors may be required for neuronal maturation and survival in the striatum.

The main focus of this study was the analysis of the survival of newborn neurons in the OB, the physiological target region of SVZ-derived neural precursors. In healthy animals, the first newly generated neuroblasts arrive in the OB after two days: by 14 days the vast majority reaches either their final destination or undergo apoptosis [10]. Previously, a reduced number of BrdU-labeled cells was shown in the RMS or the OB of R6/2 mice three or seven days after injection [20,38]. The period of 28 days after the first BrdU application comprises nearly all labeled cells arriving in the OB. These newly generated mature neurons usually integrate into the existing network of the OB. We observed that numbers of newly generated OB neurons in the GCL and GLOM were reduced by 33% and 50%, respectively, in 9-week old R6/2 animals. There are different potential explanations for the reduced number of new neurons in the OB. A decreased migration speed was proposed by a recent study of RMS neuroblasts in R6/2 mice, because of a shift in the number of BrdU-labeled cells from the rostral to the caudal portion of the RMS [38]. Alternatively, the reduction in OB neurogenesis may be related to an increased cell death in HD mice. Previously, we estimated a 50% cell survival rate of SVZ-derived newly generated neurons in the adult OB [12]. Using TUNEL staining in the GCL of R6/2 mice, we conclude that increased cell death plays an important role in the reduction of newborn OB neurons, comparable to other brain areas of this HD animal model like the cortex and the striatum, where increased cell death occurs [29]. We hypothesize that progressive dysfunction is caused by mutant htt rather in mature OB neurons and not in DCX-positive neuroblasts. Moreover, mutant htt may interfere with the integration of newly generated neurons into the preexisting neural networks in the GCL and GLOM, leading to increased cell death of developing OB neurons. Nevertheless, we can not rule out an early toxic effect of mutant htt in monomeric or oligomeric form, as the used htt antibody mainly binds to htt aggregates and is not able to distinguish different forms of transgenic mutant htt.

Further analysis of newly generated mature neurons in the GLOM revealed a shift to a dopaminergic phenotype. While most of the GLOM neurons are GABAergic, a small portion of these cells co-expresses TH [39]. Our finding that the percentage of TH-positive new neurons was increased in HD animals, was unexpected, but may indicate that the survival of these cells is less affected by mutant htt in comparison to GABAergic neurons. This further supports that mutant htt differentially affects various neuronal phenotypes differentially in the HD brain [40].

## Conclusion

Taken together, OB neurogenesis was severely affected in the R6/2 animal model of HD. Unchanged SVZ proliferation and the absence of aggregates of mutant htt in migrating neuroblasts suggest that the impact on new neurons occurs at later stages of neural maturation. While there are some indications of olfactory deficits in HD patients [4,41], it remains unclear whether the reduced OB neurogenesis may be related to this deficit. Moreover, despite the supposed increase of neuroblast migration from the SVZ into the striatum of R6/2 HD mice, the hostile microenvironment of this region lacks the appropriate signals to support mature neuronal differentiation.

## Methods

### Animals

Female B6CBAF1/J mice transplanted with ovaries from female B6CBATg(Hdexon1) 62Gpb/1J mice (R6/2, [22]) were bred to male B6CBAF1/J mice. All animals were obtained from Jackson Laboratories (Bar Harbor, USA). Tail DNA was used for determining the CAG repeat length in transgenic animals using a polymerase chain reaction assay [22]. Female mice wild type (WT; n = 6) and transgenic (R6/2; n = 6) mice were kept in normal light-dark cycle of 12 hours and had free access to food and water. Labeling of replicating cells was performed by intraperitoneal injection of the thymidine analogue BrdU (Sigma, Steinheim, Germany) at 50 mg/kg of body weight using a sterile solution of 10 mg/ml of BrdU dissolved in a 0.9% (w/v) NaCl solution. The BrdU injections were performed daily for 5 consecutive days at 5 weeks of age. Animals at 9 weeks of age were deeply anaesthetized using a mixture of ketamine (20.38 mg/ml), xylazine (5.38 mg/ml) and acepromazine (0.29 mg/ml). Transcardial perfusion was performed with 0.9% (w/v) NaCl solution, followed by a 4% paraformaldehyde, 0.1 M sodium phosphate solution (pH 7.4). Brains were removed and post-fixed in the paraformaldehyde solution overnight at 4°C. Tissues were then cryoprotected in a solution containing 30% (w/v) sucrose and 0.1 M sodium phosphate (pH 7.4). Brains were cut into 40 μm-thick sagittal sections using a sliding microtome on dry ice. Sections were stored at - 20°C in cryoprotectant solution (ethylene glycol, glycerol, 0.1 M phosphate buffer pH 7.4, 1:1:2 by volume). All experiments were carried out in accordance with the European Communities Council Directive of 24 November 1986 (86/609/EEC) and were approved by the local governmental commission for animal health.

### Immunostaining

Immunostainings were conducted as described previously [16]. For BrdU and PCNA labeling, tissue was pretreated with formamide and HCl to denature DNA. Free-floating sections in Tris-buffered saline (TBS; 0.15 M NaCl, 0.1 M Tris-HCl, pH 7.5) were treated with 0.6% H_2_O_2_. Following extensive washes in TBS, sections were blocked with a solution composed of TBS, 0.1% Triton X-100 and 3% donkey serum (Sigma). This buffer was also used during the incubation with antibodies. Primary antibodies were applied overnight at 4°C. For chromogenic immunodetection, sections were incubated with biotin-conjugated species-specific secondary antibodies followed by a peroxidase-avidin complex solution from the Vectastain Elite ABC kit (Vector Laboratories, Burlingame, USA). The peroxidase activity of immune complexes was detected with a solution of TBS containing 0.25 mg/ml 3,3'-diaminobenzidine (Vector Laboratories), 0.01% (v/v) H_2_O_2_, and 0.04% (w/v) NiCl_2_. Sections were put on Superfrost Plus slides (Menzel, Braunschweig, Germany) and mounted in Neo Mount (Merck, Darmstadt, Germany). For fluorescence immunodetection, sections were washed extensively and incubated with fluorochrome-conjugated species-specific secondary antibodies for 2 h. Sections were put on slides and mounted with Prolong Antifade reagent (Molecular Probes, Eugene, USA). Light microscopy images were obtained using a Leica microscope (Leica, Wetzlar, Germany) equipped with a Spot™ digital camera (Diagnostic Instrument Inc, Sterling Heights, USA) and fluorescent images were obtained using a confocal scanning laser microscope (Leica TCS-NT).

The following antibodies and final dilutions were used. Primary antibodies: rat anti-BrdU (1:500; Oxford Biotechnology, Oxford, UK), mouse anti-PCNA (1:500), goat anti-DCX (1:200), goat anti-DARPP-32 (1:250; all from Santa Cruz Biotechnology, Santa Cruz, USA), rabbit anti-GFAP (1:1000; Molecular Probes, Eugene, USA), sheep anti-EGF-receptor (1:100, Upstate, Lake Placid, USA), rabbit anti-calretinin (1:500; Swant, Bellinzona, Switzerland), sheep anti-tyrosine hydroxylase (TH; 1:500), mouse anti-NeuN (1:500) and mouse anti-mutant htt (1:500; all from Chemicon, Temecula, USA). The mouse anti-huntingtin antibody MAB5374 detects N-terminal mutant human htt with a repeat length > 82, in particular as htt aggregates, and much weaker soluble forms, respectively. Secondary antibodies were donkey-derived, species-specific and conjugated with Alexa 488, Alexa 568, Alexa 660 (1:1000, Molecular Probes) or biotin (1:500; Jackson Immuno Research, West Grove, USA). For labeling cell nuclei, ToPro-3 (1:2000; Molecular Probes) was diluted in TBS and applied directly to the sections for 10 min.

To determine cell death in the OB, the TUNEL assay was performed using the Apoptag In Situ Cell Death Detection Kit (Intergene, Purchase, NY, USA). The modified protocol for free floating sections was administered which was previously described in detail [42]. For chromogenic immunodetection, the identical protocol using peroxidase activity as above was used.

### Counting procedures and microscopy

All morphological analyses were conducted on blind-coded slides. Every sixth section (240-μm interval) of one hemisphere was selected from each animal and processed for immunohistochemistry. To analyze cell survival after 4 weeks in the GCL and GLOM, the number of BrdU-immunopositive cells was determined using the counting procedure described by Williams and Rakic [43]. Results were multiplied by 6 to obtain an estimate of the total immunopositive cell numbers. Total numbers of labeled cells within the GLOM were counted after determining the region of interest. BrdU-positive cells within the GCL were estimated with the optical dissector method [44] using a virtual counting frame of 50 × 50 μm spaced in a 300 × 300 μm counting grid. Labeled cells which intersected the uppermost focal plane (exclusion plane) or the lateral exclusion boundaries of the counting frame were not counted. The stereology system finally calculated a precise estimation of the total number of BrdU labeled cells within the whole thickness of the OB as decribed previously [45]. To quantify striatal neuroblasts, DCX-positive cells were exhaustively counted within the whole caudate/putamen region bordered by the corpus callosum, the RMS, the ventricle and the SVZ as well as the accumbens nucleus [45]. Total numbers of PCNA labeled cells within the SVZ were counted after determining the region of interest. All counting procedures and measurements of reference volumes were conducted on a light microscope (Leica) equipped with a semi-automatic stereology system (Stereoinvestigator, MicroBrightField, Colchester, USA) as previously described [45]. All extrapolations were calculated for one hemisphere and should be doubled to represent the total brain values.

To determine the frequency of neuronal differentiation of newborn cells, every sixth brain section (240-μm interval) was stained for BrdU and NeuN by immunofluorescence and examined using a confocal laser microscope (Leica TCS-NT) equipped with a 20× PL FLUOTAR oil objective (0.75 numeric aperture) and a 40× PL APO oil objective (1.25 numeric aperture) and a pinhole setting that corresponded to a focal plane of 2 μm. In the GCL, 100 BrdU-labeled cells per animal were analyzed for neuronal differentiation. BrdU-positive cells were characterized as solely BrdU-positive (newborn cells) and BrdU+/NeuN+ double-positive cells (newborn neurons). In the GLOM, 50 BrdU-positive cells per animal were counted for neuronal differentiation, identifying BrdU+/NeuN+ newborn neurons and newly generated neurons with a dopaminergic phenotype (BrdU+/TH+). Further fluorescence images labeling htt in combination with various other markers were also obtained on the confocal scanning laser microscope (40× PL APO oil objective, pinhole corresponding to a focal plane of 2 μm).

### Statistical analysis

The data are presented as mean values ± standard deviations (SD). Student's t-test was used to test for differences in BrdU and DCX cell numbers, BrdU/NeuN and BrdU/TH percentages, total number of BrdU labeled neurons and total number of BrdU-labeled dopaminergic neurons. Statistical analysis was performed using Prism (Prism Graph Pad Software, San Diego, USA). Differences were considered significant at p < 0.05, unless otherwise indicated.

## Abbreviations

BrdU: bromodeoxyuridine; CNS: central nervous system; DARPP-32: dopamine- and cAMP-regulated phosphoprotein-32; DCX: doublecortin; DG: dentate gyrus; EGF-R: receptor for epidermal growth factor; GAD67: glutamic acid dehydrogenase-67; GCL: granular cell layer; GFAP glial fibrillary acidic protein; GLOM: glomerular layer; htt huntingtin; LV: lateral ventricle; NeuN neuronal nuclei; OB: olfactory bulb; PCNA: proliferating cell nuclear antigen; RMS: rostral migratory stream; SVZ: subventricular zone of the lateral ventricle; Str striatum; TBS: tris-buffered saline; TH: tyrosine hydroxylase; TUNEL: terminal deoxynucleotidyltransferase- mediated dUTP: nick-end labeling.

## Authors' contributions

ZK carried out the experiments, participated in the design of the study and drafted the manuscript. MR carried out experiments and drafted the manuscript. RA carried out animal and immunohistochemical experiments. MK participated in the design of the study and carried out experiments. BW participated in the design of the study and drafted the manuscript. LA conceived of the study, and participated in its design and coordination and helped to draft the manuscript. JW conceived of the study and its design and drafted the manuscript.
